# A Survey of Middleware for Sensor and Network Virtualization

**DOI:** 10.3390/s141224046

**Published:** 2014-12-12

**Authors:** Zubair Khalid, Norsheila Fisal, Mohd. Rozaini

**Affiliations:** UTM-MIMOS Centre of Excellence in Telecommunication Technology, Faculty of Electrical Engineering, Universiti Teknologi Malaysia, 81310 UTM Johor Bahru, Malaysia; E-Mails: sheila@fke.utm.my (N.F.); mohd_rozaini@yahoo.com (M.R.)

**Keywords:** sensor virtualization, network virtualization, middleware design, WSN

## Abstract

Wireless Sensor Network (WSN) is leading to a new paradigm of Internet of Everything (IoE). WSNs have a wide range of applications but are usually deployed in a particular application. However, the future of WSNs lies in the aggregation and allocation of resources, serving diverse applications. WSN virtualization by the middleware is an emerging concept that enables aggregation of multiple independent heterogeneous devices, networks, radios and software platforms; and enhancing application development. WSN virtualization, middleware can further be categorized into sensor virtualization and network virtualization. Middleware for WSN virtualization poses several challenges like efficient decoupling of networks, devices and software. In this paper efforts have been put forward to bring an overview of the previous and current middleware designs for WSN virtualization, the design goals, software architectures, abstracted services, testbeds and programming techniques. Furthermore, the paper also presents the proposed model, challenges and future opportunities for further research in the middleware designs for WSN virtualization.

## 1. Introduction

Virtualization creates an environment in embedded and sensor networks, by which efficient sharing of resources, services and networks is achieved. Virtualization combines different hardware and software on a platform along with the network functionalities to control and administrate all the network resources. The goal of virtualization is to provide users with seamless access to the sensor data and efficient utilization of the resources.

One of the main objectives of the virtualization is to match the application needs in the best possible way, and to make sure that multiple heterogeneous sensor networks are managed in such a way that they ensure efficiency and quality. Virtualization hides all the physical details of the sensors and networks from the user application. User applications feel as if running directly on the hardware. It enables the users, services and applications to interact with each other. Virtualization enables sharing of resources in a secure and energy efficient manner [1]. Sensor networks are required to dynamically collaborate and facilitate different applications, on the air integrating sensor networks, forming clusters and supporting multiple radios. Virtualizations of networks and sensors have wide application areas that include health care, smart space, structural monitoring, telemedicine, vehicle monitoring, agriculture, industrial, military, environment, smart home, smart city and entertainment.

Sensor networks have usually been explored in application specific areas. But for the Internet of Things (IoT) to be a reality and further, the idea of IoE to be practical research community has to come up with middleware designs that have the capabilities of integrating all the sensors having heterogeneous nature and specifications with respect to power consumption, processing time, memory and transmission range.

Due to the owner's strict control over the WSN domains, conflicting goals, different sensor node and vendors, with no standard in place it is difficult to introduce a large scale federated WSN [2]. Virtualization of sensor network can facilitate a number of infrastructure providers and service providers, enabling them to combine together and support each other's interest and facilitate the user at the maximum. Virtualization also plays its part in the making sensor-as-a-service (SaaS) by facilitating both the infrastructure providers and the service providers. Virtualization decouples the functionalities in WSN. Furthermore, it opens new horizons for improvement by dividing of the traditional service providers into two infrastructure providers and service providers. Infrastructure providers are responsible for the management of the hardware or the physical infrastructure and Service providers responsible for the software, resource management and the service provisioning. The decoupling of the infrastructure from services is done by the middleware, where services are not concerned with the infrastructure and on the other hand infrastructure has nothing to do with the services.

The network and sensor virtualization can be achieved by the middleware layer. Middleware sits between the application layer and the lower layers providing abstraction from the network details. Middleware is a software tool that helps in hiding the complexities of the underlying heterogeneous hardware, radio technologies, networks and low level software. Furthermore, Middleware provides ease of managing the network resources using the concept of virtualization of the network and the virtualization of the sensors. This paper is aimed at surveying the available middleware that support virtualization, both in terms of network and sensors. A number of design approaches have been bidden for the pooling and provisioning of sensed data satisfying different functionalities and applications. The overall goal of making sensed data to be available, and to fulfill the concepts of SaaS regardless of what conditions are beaning faced at any time and place [3,4].

**Figure 1.**
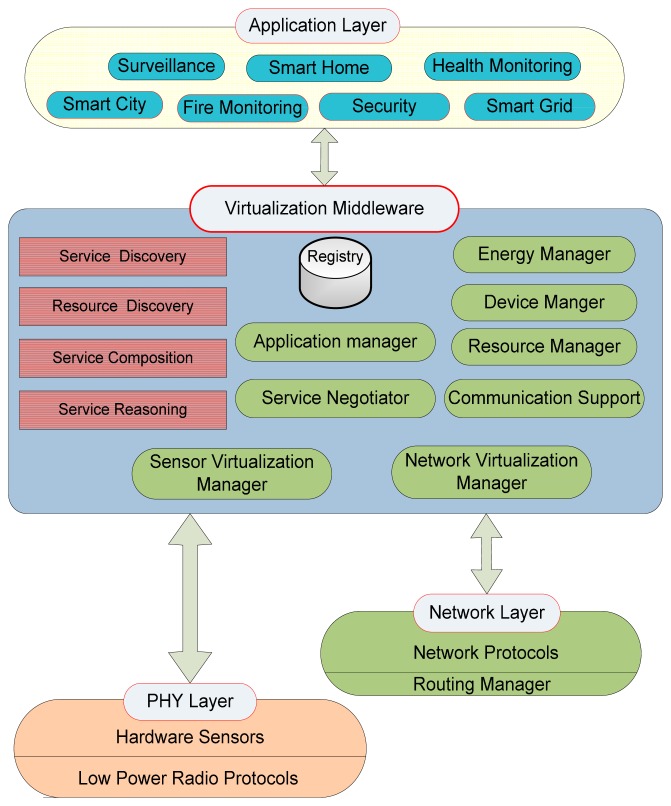
General model for WSN virtualization middleware.

Even though middleware is a well-established study area, but virtualization passes up more challenges to the sphere of middleware research. The traditional middleware techniques cannot be directly applied to all the applications because of the application's demands and heterogeneity in all respects such as devices, software and network protocols. However, virtual sensor environment can ensure the coexistence of heterogeneous WSN architectures that are unbounded from the confines of the existing multi sensor networks [5]. Figure 1 shows the general model of WSN virtualization, representing communication stack having multiple heterogeneous sensors and radio networks and network protocols. Furthermore, the slice under concentration, the middleware layer is composed of a number of modules facilitating the lower layers as well as higher layer. It acts like a bridge between the multiple sensor networks and multiple applications.

The middleware should be lightweight for the tiny sensors and embedded devices as they have memory, processing and energy constraints like TelG Mote, UC Berkeley Motes, Mica, Rene, and SunSPOT, TelosB, iSense *etc.* and should accommodate diverse communication protocols (Zigbee, WiFi and Bluetooth). Eventually the requirement of WSN middleware is to be energy aware, utilize less memory and processing.

Therefore, the aid of an efficient middleware platform is required to tackle all these wide collection of issues regarding functionality, technology, heterogeneity of devices and services [6]. A complete system collapse may happen and can cause extreme or fatal sequels for the users, especially in the health care and military scenario.

There have a been number of noteworthy reviews and surveys in the field of WSN focusing on challenges and characteristics of wireless sensor such as routing protocols [7,8], WSN [9], WSN security [10], devices in WSN [11,12], Major applications [13] and particularly in our field of interest the middleware of sensor networks has been reviewed in context of pervasive computing [14], context aware web services [7], service oriented middleware [15], context aware middleware [16], Internet of Thing (IoT) middleware [17], middleware for robotics [18], *etc.* according to the best of our knowledge, there is only one survey paper on virtualization of sensor networks [2] that only covers few of the projects and prominences on the business model and applications supported by virtualization of sensor networks. There is still a gap in the literature that is to be filled regarding the middleware capable of supporting virtualization in both respects: the sensor virtualization and the network virtualization.

The current paper provides a comprehensive review of the design, characteristics, applications, testbeds and properties of the current middlewares. Middleware contributes significantly in the field of virtualization of sensors as well as the virtualization of networks. Furthermore, our classification model is based on the middleware providing sensor virtualization and network virtualization, multi radio support.

The remainder of the paper is organized as follows: Section 2 presents the types of virtualization in WSN, while Section 3 categorizes the middleware for WSN virtualization. Section 4 shows the programming approaches for middleware virtualization, Section 5 elaborates the evaluation criteria and design goals. Section 6 discusses the abstracted services and the role of middleware virtualization. Section 7 explores the evaluation and testing of middleware based on virtualization support. Section 8 takes up the challenges of the middleware design. The proposed model is followed by the conclusion in Sections 9 and 10 respectively.

## 2. TYPES of Virtualization

### 2.1. Sensor Virtualization

Sensor virtualization is a technique that enables multiple applications to operate in a virtual environment, and isolate applications from the hardware. The isolation is made by the middleware creating multiple logical instances of the physical sensor node. Multiple instances of a sensor node are made according to the capabilities of the sensor node, *i.e.*, the service provisioning, processing power, memory on board and radio support. WSN virtualization aims to accommodate multiple logical network instances over a single physical network infrastructure with the ultimate goal of supporting applications with different requirements both in terms of nodes and communication functionalities. Furthermore, the network resources are utilized in a cost effective and efficient way [19–24]. Physical or actual sensing is attained by the direct measurement of the physical phenomena. However, it is not necessary to gather information from a particular physical sensor it can also be an intimating contact sensor. This reduces the fault in the measurement and minimizes the errors [25].

**Figure 2.**
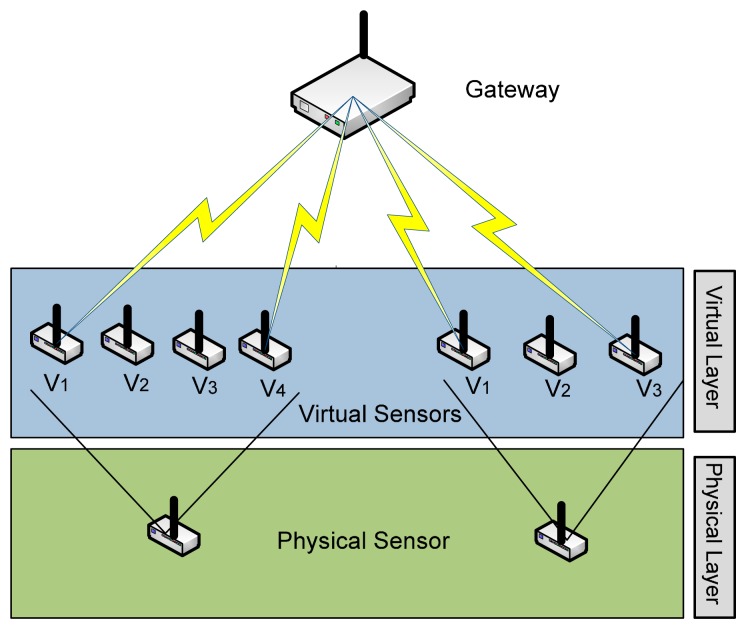
Sensor virtualization and virtual sensors.

A virtual sensor emulates a physical sensor, or it could be expressed as a software sensor that hides the hardware sensor. Virtual sensors provide indirect measurements of data by combining data from different heterogeneous physical sensors in order to provide services to the user [3]. Figure 2 shows physical sensor and the virtual sensor layer. A physical sensor can have multiple virtual sensors; however, it depends on the physical parameters of the sensor node, like processing power, on board memory and sensing capability. Virtual sensors layer lies on top of the physical sensor nodes. Furthermore, it decouples applications from the physical sensors, by running the applications on the virtual sensors.

Physical sensors and coupled hardware are costly; they are sometimes erratic and need to be maintained. Moreover, physical sensors cannot be deployed on every site that needs to be sensed. Furthermore, virtual sensors' another advantage is that the sensed data at one location can be used to predict the current and future conditions of another location. The areas already deployed with the sensors can be monitored by the real time data monitoring, the same data when transmitted to the server is then processed to predict the situation in the areas not having sensors. The example in this regard is the flood and traffic monitoring systems. The sensor virtualization technique allows the user to obtain preferred and precise information in a more efficient manner from a limited number of sensors. Furthermore, this helps in reducing the energy consumption and cost of the overall network.

Virtual sensors have a number of advantages in scenarios like replacing or maintaining a sensor node. Virtual sensor provides continuous data to the user, although the sensing mechanism of the node is discrete, and is at high time intervals in order to save the battery life. Furthermore, predicting output using techniques like artificial intelligence, in case of a physical sensor malfunctioning and failure. Moreover, virtual sensors provide significant advantages as they can facilitate multiple applications [26]. Using this approach, a better administration at node level and enhanced usability can be achieved by an increased number of different end users gaining access and control in sensors' information according to their needs and goals.

**Figure 3.**
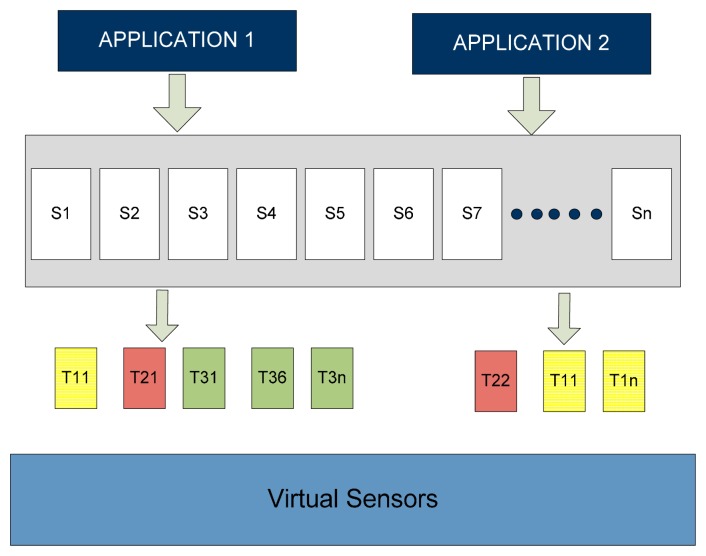
Sensor virtualization: multiple applications using multiple services.

The virtual sensor network formed may use any radio protocol. Virtualization brings flexibility in network administration, reconfiguration and scalability. Furthermore, virtualization of sensor network brings a high level of trust and security by logically separating different sensor and imposing security policies [27]. Figure 3 shows the multiple applications like *application1* and *application2* that can be facilitated by a number of services like *S1*, *S2*, *S3*, ……., *Sn*. Middleware facilitates applications by binding them with any of the required services. Moreover, the services' tasks can run on multiple virtual sensors. For example, Task *T1* belongs to *S1* bound with *application2* running on distinct virtual sensors.

All these properties stated above facilitate multiple applications to run on the single sensor node or a group of sensing devices. Furthermore, Middleware separates the specification of the sensing task from the sensing behavior and allows the developer to program the behavior of virtual sensor. Moreover, providing ease of programming and without taking care of details of construction that are to be specified in the underlying layer. Middleware enables programming of virtual sensors in an error free, secure and energy efficient manner [28].

### 2.2. Network Virtualization

Network virtualization is based on the virtual sensors and virtual links that allow multiple networks to coexist on the single physical platform. Sensor nodes can hardly perform any useful task if they are left isolated it is the overall collaboration between a number of devices that allows the system to accomplish a higher-level goal. Virtual networks can be formed by a subset of the physical network [27,29]. Virtualization of sensor network could be the collaboration of different WSN using multiple radios. It is formed by the subset of versatile sensor nodes of the sensor network, with the subset or group of sensors being committed to certain tasks or facilitating certain application at a given time [29]. Network virtualization allows a subset of sensor nodes to execute an application, while at the same time another subset of sensor nodes execute a different application [1]. These subsets vary in number, size and processing capabilities with heterogeneous PHY and MAC layer protocols according to the application requirements. Network virtualization either combines many networks to act as one or shows one network to act as many in order to meet the service requirements of the users.

Furthermore, as there are many stakeholders in WSNs with everyone aiming to achieve its goal thus heterogeneity has emerged as significant setback. However, on the other hand heterogeneity provides enhanced energy and communication capabilities [30,31]. Network virtualization promotes innovation and combination of heterogeneous node and radios. Moreover, sensor network virtualization opens new domains for business in this world of economic recession facilitating trading of sensor's infrastructure and network resources among different service providers [32].

Network virtualization decouples both the Infrastructure provider from the traditional Service provider; thus virtualization provides flexibility and QoS opens new domains for business. The resources from the multiple infrastructure providers are aggregated on a single platform to facilitate the user with diverse application using a single service provider.

Service providers can offer different services in a dynamic way on the virtual networks based the leased physical infrastructures, that is provided by the multiple infrastructure providers [27]. Furthermore, in the scenario of sensor networks the concept of sensor network virtualization is applied to separate service providers from the infrastructure providers to provide maximum flexibility, scalability and efficient utilization of resource. To achieve all this, research area for sensor network virtualization is still to be explored. Several challenges in the form of management and operations are being faced and the researchers round the globe are trying to solve these issues in an efficient way.

Virtualization provides a platform upon which innovative sensor network architectures can be built, tested and assessed [21,27]. Network virtualization enables different multiple service providers to dynamically make multiple networks using multi radios that exist together, however separated from each other [27,33].

**Figure 4.**
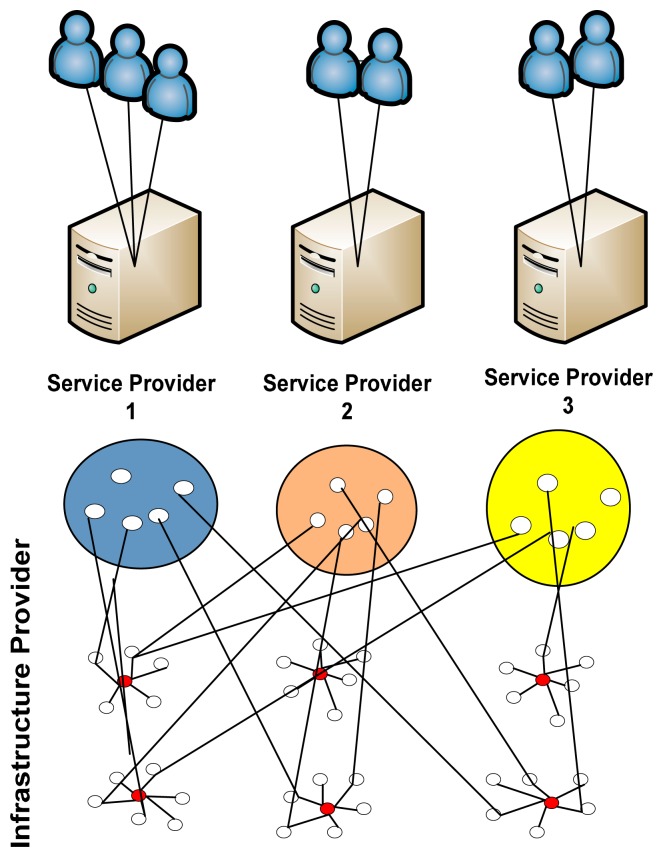
Network Virtualization.

Figure 4 shows multiple virtual sensors forming clusters based on the application demands. Each sensor is equipped with multiple radios. The infrastructure providers are responsible for the sensors and communication hardware. The service provider's job is to facilitate applications and user demands. The multiple infrastructure providers and service providers combine under SLA to facilitate users and applications. Furthermore, it shows multiple infrastructure providers and service providers running multiple applications and facilitating multiple users.

#### Multi Radio Support

The communication part is the one consuming maximum energy in a sensor network [34,35]. In order to achieve IoE low power transceiver and microprocessor system designs are critical [36]. Wireless radio choice is one of the most critical choices in the design parameters of WSN. The wireless radio is responsible for communicating among the nodes, and between the nodes, and the gateway. Traffic load in WSNs is usually low and therefore the radio does not need to be active all the time.

Most of the time the sensor's radio is in sleep mode. The radio includes idle listening mode and the actual data transmission/reception mode [35]. Radios are switched on only for a particular time when data transmission or reception is required otherwise they are kept off in order to save the energy.

In network virtualization the choice of selecting the radio is dictated by the application types and needs. The data signal transmitted from one place can face many hazards like reflection, attenuation, path loss and fading that can cause errors in the sensor's actual readings. Network virtualization allows multiple radios with different channels, bandwidth and properties to provide a wide range of options [37].

The most commonly used frequency band in WSN is the industrial, scientific and medical [38,39] radio bands. In ISM band there are a number of channels and radio choices e.g., 802.15.4 and 802.11. Furthermore, 802.15.4 is a low power radio that can offer adequate data rate up to 250 Kbps. However, on the other hand 802.11b consumes more power, but provides high data rate up to 11 Mbps [40].

There are a number of bottlenecks and tradeoffs in using more radios in a network. The difference between the energy consumption of two radios is that the energy per bit of 802.15.4 radio is 979 nJ/bit, and for 802.11b it is 112 nJ/bit, that is approximately 9 times higher than 802.11b, but when packets are formed and sent the energy consumption 802.15.4 radio is always less than the 802.11 radio [41,42]. Moreover, 802.11b have a long startup time as compared to 802.15.4 that further consumes more energy. It is an energy efficient approach to transmit a large number of bytes in a single packet so that the overhead of constructing the packet and then sending are minimized. The startup time for 802.11 is to be reduced, to take full advantages. One hop of 802.11b equals 2–3 hope range of 802.15.4.

Radios supporting high bandwidth are energy efficient only if large numbers of bytes are to be transmitted. Processors of the sensors have to play their role in using multiple radios. WiFi nodes can operate for a longer period of time with a strict energy budgeting as in as 802.11g that can operate in a power saving mode, keeping the wireless node in sleep for the most of the operating time [43]. The low power processor can't support the high bandwidth as the processor is slow. However, on the other hand, if more powerful processor is used, it consumes more energy even for the low power radio that can be operated on lower power processors [38,41]. Combination of radios and processors is vital for energy efficient, higher bandwidth transmissions [41].

Network virtualization, middleware ensures to take the maximum advantage from multiple radios, and provide QoS for the application and users.

## 3. Middleware for WSN Virtualization

Middleware is a software layer that helps in hiding the complexities of the underlying heterogeneous hardware, radio technologies, and networks from the applications as well as application developer. Furthermore, Middleware provides ease of managing the network resources using the concept of virtualization of the network and the virtualization of the sensors. One of the main objectives of the of the middleware supporting virtualization is to match the application needs in the best possible way, and to make sure that multiple heterogeneous sensor networks are managed in such a way that they ensure efficiency and quality.

As there is no standard of middleware for WSN, every research group has derived its own definition of WSN virtualization. However, a number of design approaches in middleware have been attempted for the provisioning of the sensed data satisfying different functionalities and applications.

Middleware provides an abstraction to the programmer by making a layer on top Operating System (OS). The abstraction layer provides ease of programming to the programmer. The abstraction layer defines how much low level details are to be shown to the programmer. Moreover, the more detailed programming elements if shown to the programmer the more difficult the programmer's job becomes and the more efficient the network programmed. On the other hand, if less details are exposed less the programming overhead and less efficiency. Therefore, there should always be a balance on how much details are to be exposed.

The resource and service discovery is an important and compulsory component of the recent middleware designs. Resources and services play anchor's role in WSN, all the resources are to be used in such a way that they don't exhaust early. By the resources we mean the number of particular sensing devices, the battery power left in the nodes, available radios, vacant channels, and the available memory. In WSN resources and services are dynamic; they may leave the network or join the network. Therefore, service and resources should be tracked and updated by the middleware. Furthermore, the middleware should dynamically allocate resources to users and applications so as to increase efficiency. There should be a threshold, for how long a particular resource will be reserved and serving the application. Static allocation may cause wastage of the resource and reduce the scalability of the network. However, static allocation ensures the QoS and reliability of the service provisioning.

WSNs are becoming diverse day by day due to their diversity and advancement in technology. It is essential that the middleware software architectures should be modular in order to be updated and reprogrammed with ease and to cope with technology emergence. The modular middleware design is reconfigurable and the granularity decouples the tasks, services and applications as the bases of the SOA. There have been a number of projects based on middleware for WSN virtualization. This has been a popular topic among the research community. In this section we summarize a few of the middleware supporting sensor and network virtualization.

### 3.1. Middleware for Sensor Virtualization

VITRO [44] aims to propose a service provisioning framework based on middleware that is scalable, flexible, easy to use and energy aware for the virtual sensor network platforms. VITRO is based on adaptable middleware that allows dynamic management of services and isolation between the applications and services. VITRO provides the holistic view of the network from the sensor to the user application involving gateway and core network. In the project efforts have made to stipulate a novel service provisioning framework and mechanisms for integrating heterogeneous sensors, and networks. Multiple WSN islands are formed by the sensor nodes. Different types of islands aggregate on a single point using the respective island gateway. The virtual sensor network (VSN) manager provides interfaces to the users, wireless sensor network islands (WSI) and service registry that is the database of the core framework.

VITRO's middleware layer resides on the sensor node and main modules of the middleware are: node virtualization manager, service discovery, resource discovery and energy manager. Middleware also has an interface with the node virtualization manager. Security manager also lies at the node that collaborates with the network and the MAC layer. VITRO defines the interfaces and components from sensor to virtual gateway and further, gateway connecting to virtual manager to the user applications. The session establishment sequence in VITRO shows the application negotiates with the VSN manager, VSN connects to the virtual cognitive gateway that finally connects to the sensor node. The virtualization manager uses publish/subscribe mechanism with the virtual gateway and sensor nodes.

VITRO extends the concept of virtualization from user query down to the sensor node [45]. It combines different services provided by the sensors to serve applications that provide adaptability and flexibility. The software architecture of the middleware is based on the Service Oriented Architecture (SOA) and Resource Oriented Architecture (ROA). The hybrid design is taking the advantage of SOA on the application part and ROA on the sensor part. Further, it takes into realization both the network virtualization and sensor virtualization, VITRO opens new opportunities for business by providing its service provisioning business model according to the Service Level Agreement (SLA) with different WSIs and infrastructure providers.

Requirements of a smart home are increasing, as the rapidly growing elderly population of the world is increasing. This problem is to be solved by the cost-effective network models and middleware designs. VSNware [32] is the middleware whose software architecture is based on Virtual Machine it extends the work of Mate [21] and Melete [46]. The VSNware middleware supports different applications to run on the sensor network. VSNware supports sensor virtualization it that helps in the reduction of the overall cost and complexity significantly by facilitating multiple applications. The software architecture consists of a virtualization layer based on Embedded Linux, it is a multitasking OS that hides the low level details of the hardware, software, and the sensor network. The middleware design depends on the system architecture that is divided into three main layers: application level user (ALU), sensor virtualization, network service provider (SVNSP) and physical sensors. The physical sensors are the sensor infrastructure provider (SINP), a number of infrastructure providers are aggregated on the platform of SVNSP this provides an abstraction layer between the ALU and the SINP. There could be multiple SINPs and a number of SVNSPs all collaborating to serve ALU queries.

VSNware proposes business model, sensor node architecture and a mathematical model based on graph theory and middleware for sensor virtualization. Furthermore, the key components of VSNware are network management, VSNware I/O, and application management. The core of the VSNware middleware resides at the gateway. Performance evaluation shows the design reduces the cost of network, execution time and CPU usage. However, the overhead caused by the multi applications on memory and, the lack of energy awareness of the design needs more attention.

At the University of Cambridge the Federated Secure Sensor Network Laboratory (**FRESNEL**) carried a project that includes monitoring social events of users in real-time. Middleware facilitates sensor virtualization. Several sensors were deployed in the users' working environment. These sensing devices monitor the behavior of the employee. The privacy issue was the main concern in social networking. **SenShare** under **FRESNEL** project at the Cambridge University takes into account the middleware running multi applications on different networks [20,47]; Instead of traditional fit for purpose sensor networks [48]. The SenShare middleware tackles few challenges regarding the management of the services, communication links of a single application running on different network nodes, and the isolation of applications within the network. SenShare decouples the applications from the network making virtual sensor network on top of the physical sensor network. Furthermore, SenShare middleware supports two applications: office occupancy and environmental monitoring in a building. An application running on SenShare can span across the whole network or selected subset of the physical network. Dynamic subsets of the sensor network are constructed through an overlay topology.

The middleware is implemented on power sensor nodes. The main concentration of SenShare is sensor virtualization. Middleware is implemented on top of the embedded Linux (v.2.6.29) OS platform. SenShare uses cross-application Hardware Abstraction Layer (HAL) as a middleware instead of Virtual Machine, which defines set of interfaces for decoupling of the sensors from the applications. Middleware supports split-phase interrupt handling technique. Furthermore, SenShare supports TinyOS applications in the shared environment. The major components of the middleware are network and sensor interface, application control. Access to hardware is made asynchronously through Multitasking OS that acts a HAL. The key challenges for SenShare is to limit the overhead of sensor I/O which is caused by the additional layer of abstraction, and the race conditions caused by applications demanding high access rate to the same source.

SenShare runtime adds application packet with an application routing header of 6 bytes. The overhead is likely to increase with the number of applications increasing. The virtualization layer causes linear overhead that grows gradually and slowly. The virtualization generated traffic is also the overhead on the network, but it gradually becomes low as the network life increases. The application cannot change the transmission radio of the interface, thus limits the access to the communication modules.

**Servilla** middleware is aimed to tackle the heterogeneity of different sensor nodes, and facilitate them to run different applications over heterogeneous nodes. Using Servilla developers can develop platform independent applications. It supports the discovery and use of local and remote services in a heterogeneous environment. Moreover, its bases lie in the modular SOA. Servilla address decoupling of the application from the hardware while considering energy saving as one of its key attributes. Servilla encourages different types of nodes in a single WSN (e.g., Imote2 and TelosB). Several devices with different hardware specifications like processing capabilities, storage and communication range should execute the task according to their strengths and weaknesses [49]. This would result in an efficient utilization of the WSN resources. Furthermore, servilla provides a platform to build applications that are long lived, wide-range and based on different tasks with respect to their scale, power consumption and complexity for both resource rich and resource poor devices in a single network. Servilla allows application logic to execute inside the network that provides high level of efficiency and in network coordination. The services are platform specific, but the tasks are platform independent. Servilla is implemented in TinyOS using NesC that provides two programming languages ‘ServillaSpec’ and ‘ServillaScript’. They provide a high level of abstraction like JavaScript.

Servilla consists of virtual machine that enables execution of application tasks on heterogeneous devices, tasks communicate via local tuple spaces like in Agilla. The other main module is the service provisioning framework that is further divided into consumers and services providers. Furthermore, the consumers consist of service finder, service scheduler and binding table: and the provider part contains service registry, remote invocator and the service discovery. They are responsible for updating the available services both at the consumer and service provider's part. In Servilla services are platform specific but the tasks are platform independent. Services are responsible for binding and invocation. To increase flexibility, the tasks having fewer matches are even bind to the services. Service discovery and binding are made energy efficient.

Servilla believes that the services that require high computation cost should be executed on resource rich devices. High computing services should only be invoked when required, for example, in structural monitoring application; damage localization service should be invoked when cracks are sensed. On the other hand normal cyclic monitoring services should be carried out by the resource poor devices like sensing the cracks. Furthermore, results shared by servilla show that increase in sensing frequency of the resource poor devices and invoking heavy services of resource rich devices only when needed resulting in an overall energy efficient network.

**PRESTO** [50] middleware is based on predictive proxy centric technique. It is based on the storage architecture for large scale sensor networks. The novelty lies in the middleware predictive technique. The database approach used ensures that the data should be processed, filtered, interpreted and cached in an efficient manner, the proposed technique is inspired by the primary work of COUGAR [51] and TinyDB [52].

There are two main data acquisition models first where the query is pushed directly to the sensor, this is more energy efficient [53] however, provides high latency, low reliability and low availability. The second method is where potentially useful data is pushed from the sensors to the sink, powerful device (gateway) or a database server [54]. The second model is adopted by PRESTO. The cleaning of the data is performed at the database server, thus providing lower latency, better interaction and reliability. The drawback of the model is that it is less energy efficient as unwanted data is always coming in at the database, therefore PRESTO varies the duty-cycling parameters to decrease the energy usage.

PRESTO middleware caches the current and the past data from the sensor nodes and use predictive techniques on the cached data. Therefore, provides complete support for the real time systems. PRESTO middleware also extrapolates the lost data, this helps in more cache hits. In case of a cache miss there is a significant processing involved in answering the query, this could lead to significant delay. PRESTO middleware rather than sending the raw data sends only the information or summary the data cache, which can further be refined on the proxy this leads to minimize the delay of the query processing. Albeit, PRESTO is effective in a number of applications, but the predictive sensing model could be inadequate while dealing with the sensitive applications like health monitoring or chemical plant monitoring.

**Mate** middleware [21] is a middleware designed based on Virtual Machine that support the non-expert programmers like civil engineers and biologists to program a sensor network efficiently without worrying about the timings, synchrony, interrupts, race conditions and memory constraints of TinyOS [21]. It hides the low level details of the hardware by providing higher level interfaces to the programmer. Mate is based on a virtual machine that shortens the complex programs and enables programmers to write the code in less than 100 bytes. Furthermore, the code is broken down into small chunks of 24 instructions; these instructions vary from basic instructions of sense like, *‘copy’* and *‘get’* to the complex instructions like *‘blez’*, *‘rand’* and *‘branch’*. Mate provides kernel boundaries that protect programmer to from making huge errors like disabling interrupts and memory overflow. Mate network is forced to be passive *i.e.*, one job at a time.

Moreover, Mate believes in the more processing at the node rather than transmitting, it is based on event driven programming. Huge tasks are divided into small chunks so as to avoid the stack overflow. Moreover, Mate hides the race condition from the programmer and provides support for heterogeneity of the sensors. The software architecture based on TinyOS, the tasks are queued and processed on FIFO bases in a pipelined fashion. Mate supports split-phase programming style and non-blocking execution. On the run programming can be efficiently done in Mate as it provides a concise representation of the program. Mate is preferable for small sensor nodes due to its less reprogramming overhead. Furthermore, the overhead of Mate execution grows with the passage of time and gets higher than the cost of installation.

**Melete** [46] is based on Mate [21] by adopting tiny script language. Melete is based on Virtual Machine, its goal is to support concurrent multiple applications on the nodes as well as on the network with efficiency, flexibility and reliability over wireless sensor networks. Melete is the enhanced version of Mate, it supports event driven high level programming and couples it with Trickle [55] that enables well-organized code decimation across the sensor network and the subset of sensor networks. Moreover, the network is divided into groups and subgroups that work in collaboration to carry out applications' tasks concurrently, rather than activating whole network, only a group of sensor nodes are assigned to tasks. Melete supports selective distribution of code by limiting the, *i.e.*, code is sent only within the region of interest, which covers the desired application code update [46]. Proactive distribution of code is infeasible due to the memory constraints, so Melete proposed group keyed method to decimate the code that is stored on the gateway. A dynamic grouping technique is used to support various applications along with the decoupling of the application from the sensor nodes.

In order to avoid the redundancy in the transmission of the code and to make sure the request is coming from the neighboring nodes lazy forwarding approach is used. The lazy forwarding approach provides the maximum time to the neighbors to make sure that they respond to the request posed by requesting node which has not yet been fulfilled by any other neighboring node. The more the requests the better it is to make sure where to send data. Melete uses a group key method instead of multicast method; the newly joined node can request code from the nearest group. The Progressive flooding technique is used to send different codes to different groups forming concentric circles, n-ring technique is used to search the interested group of nodes [56]. Furthermore, the drawback of the n-ring model is with every next attempt the whole previously searched nodes are re-searched so Melete propose a progressive flooding strategy which reduces the redundant search. Melete avoids the use of shared variables as the application object after the execution may drop the data. It also employs the programming of the sensor nodes to support concurrent applications.

**Hourglass** [57] is a middleware based on the SOA, supporting multiple applications. Hourglass considers naming, discovering, filtering, routing and aggregation of the data. It uses different circuits (connected networks) according to the services demanded by the consumer. Hourglass considers mobility of the data producer and make sure the data is delivered in a reliable manner. In case of disconnection it keeps the flow by using large buffer size, it stores data and then transmits when the connection is reestablished. This is very helpful in poorly connected entities and intermittent environment. Furthermore, it partially decouples the applications from the networks and provides a platform for different service providers to aggregate. Hourglass Circuit Description Language (HCDL) provides interfaces which are based on the XML to program the sensor network circuits based on TCP connections. The sensor networks use proxies to connect to hourglass infrastructure that is an overhead of the design.

At University of Massachusetts research group of Storage-centric Networked Embedded Systems (**STONE**) is working on the efficient storage and aggregation of data for multi-application sensor networks using NAND flash memories [50,58]. The role of the middleware is to facilitate the low power storage in an efficient manner.

In [59] the middleware is based on RESTful style, using Sensor Web Enablement (SWE). The middleware provides the extension of SWE by using a lightweight JSON data format instead of XML. Furthermore, it overcomes the deficiencies of data format and the architectural style of SWE. This extension helps in the development of virtual sensor network based on RESTful architecture using SWE. The results show that there is a significant reduction in the communication time.

Wendi takes the work of **MiLAN** middleware forward in [60], and allows multiple applications run on a single network. It provides policies and management tools for handling the networks and sensor nodes. It offers control policy for the sensor virtualization, and a group of sensors contributing to achieve the desired QoS. In MiLAN application and the network are unified in a single middleware system. Furthermore, it also provides flexibility to a number of network protocols like PAMAS, SMAC. MiLAN Middleware couples applications tightly with the network without affecting the control policies. Furthermore, it changes the network functionality according to the application needs.

**Mires** [61] is a message oriented middleware facilitating application to communicate in a publish/subscribe manner, where each node adversities its sensing capabilities. Mires concentrates on the environment-monitoring application, Mires middleware resides on top of the TinyOS and provides high level APIs supporting multiple applications. The middleware collects data from sensor nodes, aggregates it and then transmits it, in order to reduce the number of transmissions. In Mires middleware the session is divided into the following parts: establishment, announcement, subscription and publication.

**MAMA** Multi Application Middleware [62] is middleware providing sensor virtualization support, based on middleware abstraction layer that creates a virtual environment using grid computing. The middleware allows running of several TinyOS applications concurrently. Furthermore, thread based model is used for the middleware design. The cost of multiple application support is significant on the memory footprint of RAM and ROM. The delay and power awareness are also considered in the MAMA middleware design. Furthermore, overall energy consumption of the network is significantly reduced as fewer devices are deployed. Moreover the system can be made more energy efficient by treating different applications according to their QoS requirements.

In another work in [63] energy harvesting network and device simulator, based on TinyOS is made. It Integrates state-of-the-art simulators and provides an accurate measurement of the power aware WSN design. The design can be traced, tested and varied using the power state model of the design.

**WISeMid** middleware in [64] supports virtualization by providing services to the internet users it connects the sensors to the internet. It aggregates different sensor networks and concentrates on the energy consumption of the network. Middleware is based on SOA the nodes are programmed in nesC and the internet host is programmed in Java. The results show that stateless, Fire and Forget interaction model is the most energy efficient as it stops the process the instant the invocation is sent.

**SenSer** in [65] is a generic middleware framework proposed to support remote access to the sensor data. SenSer virtualizes the functionalities by considering them as services. Programming language and WSN development platform are made independent of each other. SenSer middleware framework is divided into three layers: presentation layer where WSN admin services reside, second layer is composed of filter, stream and query manager. Third and final layer contacts directly with the sensors and comprises of a network and the repository manager. SenSer framework is based on the Web Service Technology providing solution to client end heterogeneity.

At University of Virginia **SenQ** middleware [66] is a multi-layer embedded query system, based on declarative model. The middleware supports virtual sensors that can be dynamically discovered and shared. The algorithm reduces the network load and energy consumption, supporting lightweight network protocols. The middleware tackles the challenges of heterogeneity, dynamic deployment and in-network monitoring.

**SENSIE** [67] is one of the mega projects, taking the real world information to facilitate business. The middleware design of SENSIE consists of three main modules resource manager, service manager and community manager. Furthermore, application layer contains the application management module. The main concept of the design is based on sensor virtualization. SOA based architecture of SENSIE connecting to the cloud allows multiple services to facilitate multi applications. SENSI uses RESTful design. The resource directory, entity directory and semantic query resolver help user to run multi applications ensuring QoS.

### 3.2. Middleware for Network Virtualization

**SensEye** is a multitier network based on the heterogeneous camera and sensor nodes. SensEye uses the technique of multitier sensor network to overcome the problems of latency and energy efficiency [68]. The middleware ensures that each tier in the model consists of the homogeneous sensor nodes. Multiple heterogeneous camera sensor nodes having different capabilities are part of the multi-tier network. Therefore, in order to take the maximum benefits from the network, low power and less capable elements are assigned simpler application tasks to perform. The need for motion detection can be performed by a low resolution sensor and if the intruder is detected, then the high power camera could be woken up to carry the high definition recording. Using low cost sensors results in the increase of their number, but at the cost of reliability, similarly high cost sensors provide less coverage as they will be less in number but provide high functionality. Therefore, multitier allows low cost, high coverage, reliability and functionality by the combination of high and low power devices in an energy efficient manner [68].

Design principles of SensEye middleware include: To map the task to the less power consuming nodes having more resources, maximum use of the wakeup on demand to reduce the use of radio and processor, no wasteful wakeups of high power consuming sensor nodes, reduce delay and last one is to exploit redundancy and reduce the flow of redundant data.

**I-Living** is a project targeting medical assistance to the patients at home. The main focus of the project is the reliability, usability and security. Middleware playing a key role in the project enables independent parties to work together in a dependable and secure fashion, providing QoS for the critical applications. The software architecture of the middleware can handle multiple applications and communication protocols. Furthermore, middleware provides Java APIs for the application developer to use multiple services and multiple radios. Security and privacy are ensured in the middleware design, as every device in the home is authenticated using a token.

**SenseWrap** [69] architecture provides a virtual sensor for any type of physical sensor. The client is able to seamlessly discover the services using a standardized interface to the devices provided by the middleware. The interfaces are based on the UDP/TCP sockets. TCP/IP provides simplified application development. The full IP stack is very efficient, but due to the limited capabilities of the sensor nodes it is unfeasible. SenseWrap software architecture supports publish/subscribe interaction style. Its middleware is based on light weight SOA. SenseWrap abstracts the common functionalities of most sensor models from the application developer. Middleware decouples the sensors from the services, thus enabling multiple services per sensor node and further taking the benefits of multiple applications per sensor node. The SenseWrap middleware is based on Zeroconf, the networks and the sensor devices will be registered through the Zeroconf that is based on the services offered by each network and further each device keeps updated record of its own services offered. Zeroconf communicating with SOAP and XML over HTTP is rather light weight for the sensor nodes, as compared with the UPnP. Middleware is based on Java with sensor discovery, services discovery and client handler as the main components.

Aberer in [70] **GSN** middleware takes the virtual sensor approach and defines a virtual sensor as a data producer, it could be a real sensor, a camera, or any combination of real sensors. GSN is a container-based architecture that hosts virtual sensors based on declarative programming model in TinyOS. GSN targets to provide minimum or zero-programming for large sensor networks. The middleware components include virtual sensor manager which is responsible to manage the resources provided by a virtual sensor and interactions between the virtual sensors. Furthermore, the virtual sensor is coded in XML and SQL. GSN can support and integrate continuous and historical data. One of the main advantages of GSN is heterogeneity support in terms of devices as well as radio protocols.

Hui Dai and Richard Han in [71] proposed an overlay IP based network using ROA. It unifies the sensor network with the cloud instead to decoupling the resources and applications. Shu Lei in [72] provides uniform API for the heterogeneous devices. Bridge based approach is used to integrate different sensor networks over IP into one virtual sensor network. This virtual network facilitates the user to query data directly from a specific sensor node using overlay gateway based approach.

Philippe Bonnet in [53] provides a database approach to avoid the redundancy of the data in WNS. The query dictates which data is to be fetched from the sensor node. In Research Laboratory at Berkeley, Alan Mainwaring in [73] uses application driven architecture to monitor the habitat of an island. The design is application specific. Furthermore, the network is implemented using 802.11b and nodes with an embedded Linux.

The research presented D-uMiddle [74] middleware that provides solution for the problem in sharing sensor nodes among geological smart space. Furthermore, it works as a bridge that enables smooth interaction among heterogeneous devices and middleware platforms. A health care application providing data to the remote caregiver, the middleware provides an abstraction to the heterogeneity of the sensor nodes and provides interoperability with less overhead. The middleware is designed in the XML-based language, Universal Service Description Language (USDL) that provides high level API hiding the heterogeneous devices.

MiLAN [75] is a middleware platform with the features to continuously change and control the network functionality according to the application demands. MiLAN is an application oriented middleware. MiLAN makes feasible subsets of the sensor nodes, and optimizes the tradeoff between the application demands and the network resources. MiLAN takes into account multiple radios over the medical monitoring application. The reliability matrix is used to choose the set of sensors.

ROA based middleware is proposed in [76], it focuses on the devices and sensors rather than services. ROA is an ideal candidate for building universal APIs [26] for such middleware designs. It provides user with the direct access to the resources in an energy efficient way, whereas SOA based design is capable of providing more options to run multi applications. ROA uses REST architectural style tiny web services that are embedded into smart thing and are tightly coupled. In this model the resource can be directly accessed by the URI from HTTP browser.

In the paradigm of M2M communication, that is an extension of WSN, there has been significant work on the integration of networks and the development of standards in [77–80]. The importance of middleware in M2M is elaborated in [81–83]. To deal with multiple applications in M2M Riker in [81] proposes a middleware that supports network virtualization. Communication Manager Component (CMC) a M2M middleware component is proposed that manages data in up and down link, sleep schedule, energy of the network and device management [81]. The CMC block dynamically manages the base station overload, links and the machine devices. It also uses a common set of services that reduce the cost of development. Middleware allows applications to communicate with each other while accessing common communication mechanism. Applications are run over distributed devices and provided with high abstraction so as to hide the lower communication and device details. The communication and device management are performed at the middleware layer. In [84] work has been done regarding smart home ambient assisted living middleware framework that integrates multiple radios by utilizing both SOA and ROA in the design. Cluster tree based approach for the network virtualization is proposed in [85].

The Multi-set architecture in [86] is based on the integration of two concepts the switching mechanism of sensors sets within one network and middleware which is based on light weight mobile agents. The middleware is designed to manage the mobile agents triggering different applications and the switching mechanism. It switches between the applications using time division mode. It avoids the reprogramming of sensor node for each application, as it relies on transmitting long monolithic codes that result in huge power consumption. In the Multi-Set architecture the numbers of applications depend on the number of sensor subsets. All multiple applications run on a predefined logical sequence that is to be improved to meet the upcoming challenges of IoE. The moving agents always possess the overhead of the more energy consumption.

**MagnetOS** [87] an Operating System based on Virtual Machine. It provides a single system image on each node. The core of the middleware lies in migrating application components from node to node in order to reduce the communication cost and shorten the mean path length of the data packet. MagnetOS uses two techniques: NetPull and NetCenter moving the component single hop and multi hop in the network, respectively. Above mentioned techniques improve system longevity by reducing energy utilization. Using Java VM contains overhead of utilizing large memory due to unavailability of heterogeneity support.

The SENSIE middleware also provides the software architecture for integrating the real world with the internet. The architecture is based on the heterogeneous sensor nodes that integrate different devices and networks having 6LoWPAN and Zigbee. The dynamic resource creator provides virtual sensors. Furthermore, the interaction model is based on a RESTful architecture [67].

A multilayer architecture for network virtualization is proposed in [88,89], based on overlay network. The signaling and the data have different paths and the more complex operations are to be performed by the stronger nodes. The overlay architecture is designed in such a way that it can combine different physical sensor network as a single virtual network. The virtual sensor layer provides abstraction from heterogeneous OS. The design is based on CoAP for the underlying protocol.

#### Middleware for Multi Radio Support

**MiLAN** presents well defined APIs through which the application tells its desire to use high or low data rate protocol. It also has an abstraction layer that decouples the application layer from the network that issues orders to discover the available components and configure the network. The network specific interfaces convert Milan commands to specific network protocols. MiLAN supports Bluetooth and WiFi.

**VITRO** provides a framework that integrates multiple sensor networks of any type, using heterogeneous radios. Virtualization techniques in the middleware are used to virtualize the whole network such that it hides the low level details and different networks look like a single network.

**SensEye** is a multi-tier sensor network of cameras using heterogeneous camera devices in each layer. All tier 2 nodes are equipped with multi radios, *i.e.*, 802.11 and 900 MHz to communicate with other tier devices. The project concentrates more on bringing efficiency using heterogeneous sensors rather than the utilization of radios.

**I-living** provides a middleware framework on a cloud based concept of utilizing all the radios used in the home environment. The small devices using Bluetooth and Zigbee, and smart devices connected through the 802.11. Middleware API provides standard application services. An assisted living hub is a specialized device capable of connecting to 802.11, 802.15.4, Bluetooth, UWB, infrared independent devices. Supports TCP/IP stack at the hub.

**GSN** [28,70] also deals with the heterogeneity of the nodes and provides virtual sensor that hides the differences and details of the nodes by the use of a wrapper developed in TinyOS on every sensor node. It could hide any kind of data generator including 802.11b.

**MEMOSEN** [90] architecture takes into account the heterogeneous and mobile sensor and sinks into account. Furthermore, the architecture is composed of three layers that combine the cellular network terminals with wireless sensor networks, thus modeling the sensors and mobility. It is a hybrid model with mobile terminals using dual radios.

The framework of the middleware supporting multiple radios in the Machine to Machine (M2M) communication and managing multiple applications is presented in [81,84,91]. M2M framework in [81] works by creating an overlay network on top of the physical network to achieve high network performance. The overlay network is managed by the home gateway that collects the data and transmits it to the base station. Furthermore, Middleware based on virtual machines for M2M is developed using Linux platform in [92] to provide ease of programming to the developer in energy efficient way; the radio used is 802.15.4. The system architecture proposed by Starsinic in [91] is a home gateway based architecture supporting 802.15.4, 802.11, Bluetooth and Ethernet managing all the home devices on a single platform.

Table 1 provides the summary of the software architecture designs used in the middleware for WSN virtualization. Furthermore, it shows whether the middleware design supports the main components of WSN virtualization, *i.e.*, sensor virtualization, network virtualization, multi radio support and heterogeneity of sensor nodes.

In the literature most of the traditional middleware designs are targeting some specific problems along with the objective to support a particular aspect of WSN. Furthermore, most of the middleware architectures consider only single application. For example, middleware designed for health care application, *i.e.*, I-Living [94] cannot be efficiently used in vehicular networks as each domain and application have their own requirements and limitations.

Middleware is designed for different goals, like some provide high level of QoS, ease of programming, better management of resources, mobility support, reprogramming of nodes, heterogeneity, virtualization, handling large volume of data and multi radio support. Middleware uses different approaches in the architectural designs to achieve these goals. Furthermore, middleware provides different abstraction levels to the developer, according to the requirements of the application and programming expertise.

The middleware designed for handling large amount of sensed data, takes the database approaches like PRESTO [50] and middleware in references [53,58]. The middleware designed for health care services demand high QoS and has to ensure the transmission and data integrity as proposed by MiLAN [75] and [79,94]. For the mobility support and applications like fire monitoring middleware employs the mobile agent techniques using tuple space that has the ability to enter or leave the networks as used in Agilla [95]. Moreover, the middleware for a single application and multiple applications have different attributes.

From the developers' point of view, if we intend to go for ease of programming, then, declarative programming model based middlewares are selected. Declarative programming technique provides a high level of abstraction from the low level programming details. On the other hand, middlewares based on imperative programming models provide higher efficiency although are more difficult to program. However, for the heterogeneity support over the hardware and software, middlewares are focused on supporting multiple platforms like GSN [27]. The middleware aimed at the reprogramming of the WSN on the air, use small piece of codes for updating or changing the behavior of the sensor network. Middleware based on VM are introduced by Mate [21] and Melete [46] for the reprogramming WSN. In general, there is a need for a middleware design that is holistic, that provides high flexibility and covers the maximum attributes mentioned above. Table 2 provides the list of devices that are used and supported by the middleware for virtualization.

## 4. Programming Approaches for Middleware

The research in the field of efficient software development, management and deployment is still under investigation. Furthermore, there is a need to reduce computational, deployment and development overheads [42,96]. The main focus of attention is WSNs are the mW device/node with a balance computing and communications. Therefore, software designs and programming approaches for the middleware design should facilitate the tiny devices according to their computation capabilities. Ease of programming is the most important aspect of the software design and is directly related to the programming models [97]. There is always a tradeoff between developer's ease of programming and efficient network resources utilization of the network. Energy awareness, routing, efficient resource utilization and data delivery have always been the key parameters for an efficient programming model for WSN.

In network programming, whole sensor network is treated as a single abstract machine that provides a high level of abstraction. In the database inspired models, network programming approach this best suited. On the other hand it also limits the range of applications. Programming paradigms for WSN middleware can be categorized into the following programming models [97,98]. Sensor and network virtualization can be achieved using the following programming models.

### 4.1. Agent-Oriented Programming

The term Agent-Oriented Programming (AOP) was coined in 1989 by Yoav Shoham in [99]. This paradigm is helpful in programming the decentralized systems and solving their problems. It is defined by Nicholas R. as “an agent is an encapsulated computer system that is situated in some environment, and that is capable of flexible, autonomous action in that environment in order to meet its design objectives” [100]. Such paradigm fits well for WSNs due to their decentralized nature. In the agent-oriented programming model, agents are self-governed, problem-solving entities that interact with each other through a high-level communication model. The basic units of AOP are beliefs, commitments, capabilities and choices. AOP is extremely unpredictable as it deals with the dynamic environment. The communication in AOP could be successful or unsuccessful.

Agent oriented approach for the middleware designs is used in [101,102]. Agilla middleware in [95] uses mobile agents that have the capability to migrate from one node to another node in a network. Weak migration only migrates the code not the state information. On the other hand, strong migration transfers both the code and state information allowing the agent to resume execution at the destination.

### 4.2. Component-Based Programming

Component-Based Software Engineering (CBSE) [103] proposed by Szyperski in 2002 is accepted as a well-structured programming model to develop software systems. CBSE provides interface-based interactions between system components or modules and avoids all hidden interaction between the components that is via direct function calls between the components, variable access, or inheritance. This provides a high-level programming abstraction and provides capabilities to integrate modules in order to simplify configuration and maintenance of software systems [104].

One of the major advantages of component-based software development is module reusability and standard API provision. In WSN there have been some very noteworthy efforts to involve CBSE in WSN applications. There have been very few successful efforts, and are being used for developing many WSN applications.

nesC is the most popular programming language in WSN based on CBSE and certainly the most successful one [105]. nesC is an event-driven programming language for WSNs derived from the C language. It is originally proposed to develop the TinyOS operating system—the one of the most popular system software for sensor nodes. nesC is a structured component-based design for building embedded systems. Few examples of component based programming models are OpenCom [104], Think and LooCI. Spatial programming is a space aware programming model, which keeps track of the sensor node's location. Message passing like mobile agents is used to communicate between the nodes [106].

### 4.3. Event Driven Programming

The event-driven model is the most popular programming model for WSN today. In WSN programming, event based programming is a programming paradigm in which the flow of the program is determined by events. For example, the sensor outputs, threshold level cross user actions or messages from other programs or threads. Programming sequence is divided into two portions: the first one is to detect an event, and the second is how to handle the event. Event based programming makes systems to provide the quickest possible response to the events of interest.

Event driven supports more flexible and extensible architectures, as it allows components to be removed and added without consequence to the rest of the system. Event based systems Increases complexity and reduces control over the interactions of devices. On the one hand, improved flexibility and scalability is achieved. According to Chandy and Schulte, an Event-Driven Architecture stands by to the following principles [107]. Event driven coding can be done in any language, although the task is stress-free in languages that provide high level abstractions [107]. Few event driven models, include TinyOS, nesC, Contiki, ProtoThreads, DS-Ware and Agilla [95].

### 4.4. Imperative Programming

Imperative programming model computation is described in terms of a program state and the statements that change the states. The program while execution generates many states. The transition from one state to the next is controlled by data assignment operations and the sequencing commands [107]. The imperative programming model forces the programmer to write code that provides the details of the exact steps that are to be followed by the sensor nodes in order to achieve the goal of the application. This model is used in contrast to declarative programming paradigm, which is only concerned with the program output without knowing how to achieve the task. Examples in the imperative programming models for WSN are Abstract Task Graph (ATaG) [108].

### 4.5. Functional Programming

Functional programming is a programming paradigm in which the main method of computation is the application composed of functions. Functional programming involves making and manipulating functions to build up larger programs. These functions can be of different types depending upon the application's need and the situation. Functions can take input and return data, the data values or variables could be taken from the ongoing sequence of the program or from the other functions.

One of the main advantages of using a functional programming is it hides the direct interaction of program starts from the programmers. Furthermore, the functional programming in WSN is that it extracts parallelism from the manipulation of data. For instance, a function can handle streams from multiple sensor nodes and can be compiled efficiently within the network. Examples of functional programming for WSN include the Regiment middleware [109] its goal is to reduce the programming efforts for complex sensor network applications development.

### 4.6. Object-Oriented Programming

This programming paradigm is based on maintaining a unique mapping between objects of the classes the relationship between corresponding environmental elements. Object Oriented Programming (OOP) model for WSNs encapsulate data, computation, communication, sensing and actuation dynamically. Object instances are created dynamically by the model when the sensor elements are detected and are destroyed when these elements leave the environment. This also leads to the execution of program code at the location of the physical entity which is ideal for sensing and actuation tasks. EnviroSuite [110] is based on OOP model provides an abstraction layer to avoid low level details while programming WSN.

### 4.7. Message-Oriented Programming

Message Oriented programming (MOP) model exists in SOA. MOP is based on writing a program section that is generating messages and replying to the messages just as events happening like interrupts. In WSN MOP uses publish subscribe mechanism between the gateway and the sensor node. This model provides a loose coupling between the transmitter and the receiver [98]. One of the major benefits of MOP in WSN communications is that it has the ability to store, route, and manage messages while transmitting them from source to destination.

### 4.8. Set-Based Programming

The set based programming model is based on the theory of sets, borrowed from the mathematical field of study. Set based programming model in WSN is driven by the fact that sets provide in depth knowledge of the data and resources it is a natural way to think about efficient resource management in a WSN. In which, a node or union of nodes combined to carry a particular task. Programs based on set theory perform various set operations such as union, intersection and iterating over the elements of the sets. μSETL is a programming model for sensor networks based on set theory [101].

## 5. Design Goals and Evaluation Criteria of Middleware of WSN Virtualization

The designing goals of a middleware for WSN virtualization are as follows. The middleware design should meet the application demands.

### 5.1. Network Heterogeneity

Middleware design should tackle the heterogeneity in terms of hardware devices, OS, Network models and capabilities. It must provide freedom for any type of network using any PHY or MAC layer protocol to be a part of the virtual network. The middleware should allow different infrastructure providers deploying heterogeneous devices to be integrated. The hardware abstraction layer or wrapping techniques are used to address the heterogeneity issues. References [20,31,44,45,70] provide solutions for heterogeneity of networks, devices and platforms.

### 5.2. Flexibility

The middleware design for WSN virtualization should accommodate and absorb internal and external changes in the sensor networks. The service providers and the infrastructure providers should be independent of each other, such that they make changes in their policies, add/remove the software or hardware independently. The middleware design should be flexible along with the resource discovery and the service discovery modules essential for virtualization of WSN.

### 5.3. Decoupling

Decoupling of components is the key for virtualization and future IoE. Decoupling of the hardware devices, communication radios, services, networks, software modules and applications from each other is essential. These components should be addressed in the middleware design for WSN virtualization. The middleware should support the overlay networks to provide logical isolation of the hardware and software. The service discovery and the resource discovery are the modules responsible for decoupling of application from the network. There are many benefits of decoupling. However, the decoupling demands more memory and processing power.

### 5.4. Network Management

In WSN virtualization every aspect of the network is made independent and isolated. Different applications need to run on the network using multiple services and further, the services invoking a number of tasks. Moreover, multiple radios providing more flexibility to the application and adding more complexity to the network and middleware design. All the operations stated above are to be managed in an efficient manner. Furthermore, network management will improve the QoS for the applications running on heterogeneous network. The node virtualization manager and the network virtualization manager are the key modules that interact with the other components such as energy manager, resource manager, service manager to manage all the activities of the virtual sensor network [2,44].

### 5.5. Scalability

Middleware for virtualization must provide scalability, in terms of number of nodes, and also in term of number of users and services. Furthermore, the virtualization, middleware must be able to cope with more transmission load and service demand from applications and users. The middleware design must be scalable so as to provide room for large scale networks to be deployed and integrated. Multiple service and infrastructure providers should collaborate in such a way that the system becomes highly scalable.

### 5.6. Abstraction Level

The abstraction level is directly related to the ease of programming approach. The virtualization, middleware controlling the major components of the networks, each and every aspect should not be exposed to the programmer and should provide high level APIs to program the network. However, this technique has a drawback that programmers can't achieve the desired network efficiency. The middleware abstraction plays vital role in providing efficient, high abstraction for the programmer.

### 5.7. Robustness

The middleware should be robust and particularly for the sensitive applications like health care, which demands robust middleware design. This style of middleware design prevents the abnormal termination and software failure. Virtualization middleware should handle the errors during session establishment and the execution of the applications' tasks. It should provide backup for the smooth sensing operations in case of failure or system crash. Robust programming style and algorithms can result in a robust middleware. However, this is achieved at the cost of more processing and memory utilization.

### 5.8. Energy Aware

One of the major factors to care of beside the memory constraints is the energy constraint. In a virtualization environment where multiple communication radios and protocols are used, the middleware is to make sure the energy efficient radio selection is done. To achieve energy efficiency the middleware has to make virtual grouping of the services and devices such that the network longevity increases. Furthermore, the high power consuming nodes and protocols should be used when there is no other choice to meet the application demands. The energy manager module and the sensor virtualization modules collaborate with each other to minimize the energy consumption and possibly without comprising on the QoS.

### 5.9. Security

The middleware should play its part in securing the data and make sure the data is securely transmitted to a particular application. The middleware should make sure that applications like health care and the military that contain sensitive data should be accessed by the authenticated users only. The authorized access to the sensor data is the responsibility of the authentication manager module, residing at the middleware.

### 5.10. Fault Tolerant

Fault tolerance is the attribute of virtualization middleware. The middleware design should be fault tolerant, if any sensor or group of sensor dies, the dynamic regrouping capability of virtual sensor network overcomes the faults. The device manager module keeps the device parameters updated, that helps in reducing the faulty readings. The middleware should be programmed in such a way that the program is easy to debug. There should also be some recovery mechanisms to bring the network back running in case of problem.

### 5.11. QoS

Many applications demand QoS, therefore the requirements of the application must be satisfied. In virtualization specifically, processing latency, choice of service, virtual links, nodes, transmission power and radio selection are very critical all these decisions must be made in such a way that it meets the QoS requirements along with the minimum use of power. The applications like personal health monitoring, fire detection and disaster monitoring require high QoS. Virtualization of sensor networks enhances the QoS by virtual links and QoS manager a vital module in all middleware frameworks.

### 5.12. Memory Utilization

The memory of the sensor node is one of the most precious things on board. The middleware has to find an optimal point in providing all the above stated properties and the memory usage. The more code the better the performance. However, the programming techniques like modular and component based allow programmers to use memory efficiently by reusing the code and using pointers effectively. The middleware architecture should allow the programmer to control the memory regions. EEPROM should be used as a program memory. This helps in reduction of the memory footprint.

### 5.13. Processing

Processing is another constraint of sensor nodes. Usually, the sensor node's 8 bit microcontroller has less processing capabilities. Therefore, the code should be made such that it avoids the infinite loops; uses shift instead of multiplications, power and division. Use ‘unsigned int’ if possible instead of signed numbers so as to reduce the processing.

## 6. Abstracted Services and Middleware of WSN Virtualization

In order to fulfill the requirements and design goals mentioned in Section 5, the role of abstracted services is non-trivial. The middleware design for virtualization should offer these services in order to fulfill the users, services providers and infrastructure providers' demands. These services include: clustering, real-time, data aggregation, localization, security, delay tolerance and load balancing which are discussed as under.

### 6.1. Clustering

Cluster-based middleware architecture localizes the collaboration among the group of sensor nodes providing services to a single or multiple applications [111]. Middleware must be designed in such a way that coordination and control between the virtual clusters is seamless. The major clustering techniques are based on identifiers, position of node, weight of node, Residual energy of node, channel and frequency.

A geographical forwarding technique based on the virtual creation of clusters is provided in [112]. The overhead in management of the virtual clusters, excess of control signals in the network and data aggregation of the sensor nodes are the key challenges in middleware for network virtualization. From the prospective of the service provider a virtual network should be able to discover the presence and topologies of other coexisting virtual clusters for better coordination. Moreover, virtualization demands on the fly auto-configuring of clusters and inter-cluster synchronization according to applications' demand. To tackle these challenges middleware has to go deep in the cross-layers design of network and MAC layer.

### 6.2. Real-Time

For the real time scenario, a system must be dependable with respect to timing errors. Middleware needs to have the attributes of availability, reliability, safety, confidentiality, integrity and maintainability in order to fulfill the requirements of the real rime applications such as disasters and emergency surveillance [113].

The middleware for virtualization has to look into the application execution, resource management and allocation, and has to provide services to facilitate all the real time applications. Furthermore, in order to achieve real time response from the virtual sensor network, middleware has to be light weight and the algorithm must be based on multi-threading and if possible use multi-processing techniques. Middleware must apply delay as the metric to facilitate real-time applications.

A big challenge is how a middleware is going to accommodate all these properties of real-time systems, keeping in view all the constraints of the sensor network environment. It is still an open area for the research. The efficient switching between radios like 802.15.4 and 802.11, selecting the sensor nodes near to the gateway/sink, dynamically changing the response of the network for real-time applications and forming clusters based on fast processing speed could be a few solutions.

Considerable R&D efforts have been put forward on developing real-time middleware in the past by **RT-STEAM** [114] is a middleware supporting real time applications in vehicular Ad Hoc Networks. **RAP** [115] provides base for a real-time architecture for large-scale sensor networks. **CORBA** [116], **CORBA** Component Model in [117].

### 6.3. Data Aggregation

In organizing a distributed virtual network, the nodes middleware not only just pass the data or provide the services to the applications running on top; they are also involved in aggregation of the data from the sensor nodes. This technique increases the power gain of the network [118]. A number of middlewares in the literature support the data aggregation those include [51,61,119–122]. In virtualization of sensor networks the importance of the aggregation data increase as the middleware is dealing with multiple services and applications, so researches need to find new efficient techniques of data management and aggregation. The data aggregation policy should try to use a single service data for multiple applications rather than fetching the data every time from the sensor nodes. This technique is very useful for non-critical applications. However, the algorithm for data aggregation should try to minimize the error probability while aggregating the data for multiple applications.

### 6.4. Localization

In order to support multiple applications, a number of applications need to know where the services are. Localized middleware benefits by knowing the location of the sensor node, services offered by the sensor node, and power parameters of each network, cluster and their respective nodes. Therefore, by knowing all these parameters, it reduces the data transmission delay, forms cluster efficiency and reduces power consumption. There are a number of techniques used in localization one of them is a Graph algorithmic technique that is also helpful in localizing the WSN. This technique includes: polynomial-time [123], Euclidean positions and inter-sensor distances used in [124], Graph rigidity theory by applying grounded graphs in [125]. These techniques can be incorporated in middleware designs to traverse the network and get the location of the nodes and the networks.

The localization problem is quite challenging, and need considerable attention while designing a middleware framework for sensor network virtualization. Miro [126] is an object-oriented middleware designed for robots addressing the localization problem. RT-STEAM [114] is a middleware for real time applications like vehicular networks. RT-STEAM is based on the concept of localization. Furthermore, it has location identification service that uses sensor current geographical location and allows the middleware to compute the power of the signal to be transmitted efficiently. Few common algorithmic techniques used in WSN localization are Triangulation [127], Ad-hoc positioning [128,129], N-hop Multilateration [130] and GPS-free node localization [131,132].

### 6.5. Security

Federated virtual networks offer several services and multiple applications use these services on virtual sensor networks, with a number of service providers and infrastructure providers all collaborating with each other to serve the users seamlessly seems quite adorable. Future of sensor network is IoE which states that everything is connected: from personal home sensor nodes to the sensor nodes in public places like streets and shopping malls. Middlewares currently concentrate more on the resource allocations, efficient communication and management of the sensor networks. However, the security of the services, resources and data is a huge challenge.

With multiple networks collaborating, security should be a vital part of the future middleware design. The developer of the network should have more options to increase and customize security mechanisms. The flexibility, scalability and extensibility of the architecture are reduced when more security mechanisms are implemented. Therefore, to cope with these challenges, new algorithms and techniques are to be introduced with minimal effect on the flexibility and scalability of the federated virtual networks. There are a number of middlewares ensuring the security like: Proactive Code Verification [133], SMEPP Light [134] which is based on cluster management, and provides group-level security algorithm. Furthermore, it provides mechanisms for query injection and data aggregation based on publish/subscribe mechanism. Furthermore, middleware for securing mobile agents based WSN is proposed in [135]. Security mechanism based on a link-layer remote procedure call is presented in SpartanRPC [136]. A security framework for federated sensor networks is provided in [137].

### 6.6. Delay Tolerance

Delay Tolerant Network (DTN) is aimed at enabling reliable communication over links that do not offer high quality transmission. Therefore, these links provide packet loss and long delays. DTN provides a solution to these problems by applying buffers at each hop and using feedback from the destination node. Buffers keep the messages in memory when link quality is unsatisfactory, and transmit when link quality is good.

Middleware for virtualization provides the solution due to its dynamic nature. Furthermore, middleware for DTN can provide virtual links and buffers on the overlay network to improve high latency/low data rate. Furthermore, it provides solution for disconnection and transmission errors due to the knowledge of the whole network. VITRO middleware framework provides solution the DTN [44,45] by virtualization of the network. DSAM [138] is a middleware that provides a communication layer between the service and different applications for DTN. Middleware proposed in [139] dynamically adapts the connections. Moreover, it switches between the connections according to the network applications and conditions.

### 6.7. Load Balancing

The basic idea of load balancing is to share traffic load among the nodes in order to reduce the probability of error, due to queue overflow at some nodes. Load balancing middleware services improve the scalability and overall system throughput of the sensor network. However, most of the solutions are meant only for a specific application and environment. There is a need for a generic model for load balancing in network virtualization, as load balancing service doesn't work other than the application it was designed for [140].

Network virtualization allows sharing of traffic inside the virtual clusters. That is quite effective, because nodes in the same cluster have a similar goal or similar application to serve. Load balancing can be done at different levels in network virtualization environment, it could be network based, inter-cluster based, intra-cluster based, sensor based and applications based. GSN [28,31,70] for high performance tries to achieve load balancing among the different sensor nodes using default polices of the middleware.

## 7. Testbeds and Experimental Resources

In the paradigm of wireless sensor networks, testbeds and experimentation, play a significant role in the successful deployment of the sensing network. Furthermore, testbeds allow testing of all the sensing and communicating aspects of the sensor networks in a realistic setup [141,142]. Testbeds are crucial for debugging and evaluating middleware for wireless sensor networks. Testbeds play vital role in testing the behavior of embedded systems e.g., effect of temperature on sensor nodes is tested in [143].

There are number of tools that support testbed composition [142], like hardware devices provide the real evaluation of the system. However, the hardware costs and heterogeneity of testbeds formed are usually limited and provide less flexibility due to static configurations and small size. Moreover, for the IoE to be a reality we require large scale federated networks to be formed and tested. The hardware approach is usually limited to a single network and don't allow large federated networks to be tested and evaluated. The research community is working on the testbeds that allow large federated heterogeneous networks to be evaluated and tested.

The easiest technique for evaluation of middleware for sensor network is a simulation; it speeds up the development process. Simulation provides flexibility in terms of radios, sensor nodes, heterogeneity, scalability, mobility, debugging facility, OS. Furthermore, it supports large scale federated networks to be easily tested. However, it lacks the conviction due to its inaccuracy and unrealistic approach. Therefore, this technique is limitedly used for planning and deployment middleware for WSNs and systems.

There are two extremes in testing so WISEBED [141] came up with the concept of combining both the testing techniques: simulation and the hardware testbeds by virtualization of the testbeds. Furthermore, this concept provides flexibility and accuracy at the same time. Testbed virtualization can provide high efficiency in testing without hampering the realism of experiments. There are a number of testbeds for WSN that include SensLAB, moteLab, CitySense, Sensei and Wisebed having different approaches [144]. Few of the testbeds that allow evaluation of middleware based on virtualization are discussed as under.

**WISEBED** [141] uses the concept of virtual testbed (VTB) that combines the physical and simulated sensor testbeds using overlay network technology that could be used to support different physical and simulated testbeds. VTB allows large federated networks to be tested with a fair amount of accuracy. VTB provides flexibility in connectivity between any physical, simulated or emulated sensor nodes through virtual links and virtual radios, it also supports virtual mobility [145].

WISEBED has come up with the middleware based on the web service called iWSN. Middleware supports multiple OS, simulators and provides a high level API for the application development. Furthermore, it also provides the software development kit (SDK) for the researchers around the globe to design, implement, develop and synthesize the algorithms on top of the VTB. Moreover, it supports multiple OS like TinyOS [146], iSense and Lorien [147]. VTB is specified through WiseML based on XML and WiseLib algorithm library for networked embedded devices. WISEBED contain a number of heterogeneous nodes that includes Pacemate, iSense, TelosB, Tmote, MicaZ, SunSPOT, TNode, Tmote Sk, MSB-A2 distributed around the globe [144,148]. There are a total of 9 geographical sites with 550 nodes [149].

**FlockLab** [150] testbed based on the physical sensor nodes, it overcomes this limitation of the previous test beds by allowing several services to run simultaneously on all nodes. Furthermore, FlockLab provides timing information in the low microsecond range that enables events to be correlated with power samples. FlockLab also achieves a better synchronization, allowing for a better alignment of power traces recorded at nodes. FlockLab's middleware services synchronously collect huge amounts of logged data from the scattered sensor nodes. It also extracts the radio state and support network virtualization.

**SmartSantander** [151] is a unique large scale experimental research facility for the provision of smart city application. It envisions the deployment of 20,000 sensors around the city, currently there are 3000 IEEE 802.15.4 nodes, 200 GPRS modules and 2000 RFID tag/QR, and they are static as well as mobile. One of the major purposes of SmartSantander facilitates experimentation for the research community, infrastructure providers and the service providers to speed up the proof of concept, and IoE to be a reality. Furthermore, Assessment of how society responds to the IoE technologies and services [152]. **SmartSantander** middleware and software supports both the testing of sensor virtualization and network virtualization.

**FIRE** (Future Internet Research and Experimentation) [153] project provides a research platform which facilitates a number of the researchers and developers for the Future IoE concepts. FIRE provides large-scale experimentation facility that supports both medium and long term research plans. FIRE based on virtualization provides the tools that are needed to conduct large-scale experimentation on innovative paradigms of WSNs. Furthermore, a variety of network experimentation can be conducted on the given platform. FIRE facilitates testing infrastructure supporting multiple technologies like having diverse characteristics like mobility, scalability, security and privacy. Furthermore, it allows testing and evaluating the middleware based on sensor and network virtualization. Fire projects focusing on testbeds are EULER, FIBRE, RELYonIT, OFERTIE, STEER, SOCIAL&SMART, IRATI, 3D-LIVE, CLOMMUNITY, EAR-IT, ECO2Clouds, ALIEN, EVARILOS, Cityflow, IoTLAB, FORGE, TRESCIMO, MOSAIC 2B, SMARTFIRE [154].

**OneLab** [155] is the pioneer of the federation concept for the testbeds. OneLab is also a part of the FIRE project based on the heterogeneous testbeds to federate their computation, database and network resources. **PanLab (**Pan-European Laboratory) [156] is also part of FIRE project and takes the work of the OneLab forward by developing the framework for the federation of testbeds on a wide scale. PanLab Combines all the emerging testbeds and experimental resources together in such a way that tests and experiments are executed according to the demands of the emerging concepts in WSN. It is based on the VTB concept and forms Virtual Customer Testbed (VCT). VCT fulfills the requirements of the federation and customer view. Moreover, manages the underlying infrastructure elements participating in the testing/experiment execution. Teagle [157] framework provides interfaces of the Panlab federation resources, and interacts with Panlab testbed manager and manages the customers' request and the heterogeneous testbed devices. Furthermore, Panlab also provides the concept of resource adaptors that abstract the testbed manager from the offerings, and provide a single API to Teagle Web portal for the setup and configuration of VCTs.

**BonFIRE** [158,159] is a free, open access scheme that facilitates researchers for a faster, cheaper and more flexible tests on new business models. BonFIRE has developed a cost effective business model. Researchers can test a wide range of cloud experiments, such as cloud bursting and hybrid clouds, across five European sites. Furthermore, provides a federated, distributed cloud testbed based on virtual machine. VMs are located at different Clouds that federate different sites and enable seamless experimentation. The BonFIRE middleware has been designed to provide experimental support over multiple heterogeneous devices evaluating infrastructure and virtual machine level. The middleware consists of multiple layers where each layer exposes its functionality via a set of APIs.

**TWIST** [160] is an open access test bed having 204 nodes, middleware support heterogeneous devices and allows integration with Cooja Simulator. TWIST testbed provides indoor experimentation facility and consist of TelosB and eyesIFX nodes.

**IoT Lab** [161] provides a very large scale open source facility for testing and evaluating wireless sensor heterogeneous devices and software architectures. It consists of 2700 wireless sensor nodes spread across 6 different cities. IoT Lab's middleware supports virtualization and provides high level APIs for drivers, OS and libraries for programming and testing algorithms and protocols. Furthermore, IoT Lab testbed allows development of the applications and middlewares on top the infrastructure provided. Moreover, it can be testbed with or without the OS support. Table 3 shows the testbeds that support virtualization; it also shows the devices that are used by the testbed. Furthermore, the number of devices that are part of the testbed and the services offered by the testbed.

## 8. Challenges and Future Work

In WSN virtualization there are many open problems, open issues and questions still to be answered. Middleware design for the virtual sensor network has to deal with the challenges like: guaranteeing connectivity, choosing the optimal frequency, localization, data aggregation, security, load balancing, radio selection, clustering and inter cluster coordination. To achieve all the mentioned properties we need a comprehensive middleware design for WSN virtualization. To achieve this goal middleware architecture has to face several non-trivial algorithmic challenges.

In WSN virtualization, multiple applications need to run; each application has its own requirements as reliability, robustness, fault tolerance, high throughput and QoS level. The applications like elderly care, military, smart home, smart city and traffic controlling are the examples of the applications that need more reliable, stable and robust networks. Due to the limited resources of the sensor nodes and the networks, it is highly probable that the performance of multiple running applications cannot be satisfied simultaneously.

For network virtualization, middleware should provide a general runtime environment to the application developer. Furthermore, it should also guarantee the correct functionality of concurrent applications running on the multiple networks consisting of resource constrained devices. Therefore, the middleware should manage multithreads efficiently to reduce the probability of error and failure.

Middleware is required to efficiently tradeoff among the several QoS dimensions of a particular application as well as multiple applications. Therefore, a mechanism is to be derived such that it adopts the policy of tradeoffs between different QoS parameters and available resources. Software design for network virtualization should collectively fulfill the application needs.

One of the key challenges of middleware design is to make energy-aware protocols and policies that should gather the power information from the multiple physical networks and make virtual networks according to the power parameters. However, contrary to the power awareness QoS need not to be compromised. Therefore, an optimized metric for QoS parameters, applications running and available resources must be part of the middleware design. Furthermore, mobility of sensing devices imposes extra challenges to the network virtualization. It poses a number of challenges like QoS provision, clustering and localization.

There must be a standard to be followed while dealing with service and resource discovery module. The service provider should get the parametric values of the resources and services from distinct heterogeneous networks in a standard format. This would lead to efficient resource and service allocation. Moreover, the role of multi radios is quite significant in the future network designs. The research work carried out on using multiple radios is very limited, and this area is still open for the researchers and need significant improvement. Another important issue in the middleware design is data aggregation; middleware for network virtualization should provide mechanisms to merge and synthesize the data.

Network virtualization creates virtual links between nodes. The speed of the virtual link should not be too slow as compared to that of a native link. The programming model for virtualization middleware should be based on multi-threading techniques so as to speed up the process. In order to meet the demands of each application the middleware should be able to fulfill application requirements and provide services in a seamless manner. On the other hand the complexities of networks, devices and platforms are also to be managed in such a way that the network longevity is increased and the resources do not exhaust early.

- There are different types of sensors with diverse physical layer protocols, there is a need of a middleware platform on which they can combine, and coordinate to achieve the goals.
- Sensor nodes as they are tiny devices with small battery, processing power, and memory. They are prone to different failures of hardware and software, the software errors are to be minimized by the middleware software architecture.
- Ease of programming provided by the middleware to the developer should not affect the network performance.
- Middleware based on virtualization demands more memory, so the middleware design should be light weight.
- Middleware for virtualization need to discover services demanded by the user and applications in an efficient manner, so as decoupling between applications and network should be at optimum level.
- Service and resource discovery algorithms need significant improvement for virtualization middleware.
- Security of sensed data is a major challenge where multiple applications accessing different services and the sense data where as simple middleware has only one application to serve.
- The middleware design for virtualization should support cost effective business models as it opens new horizons for investments and business opportunities.
- Unlike conventional middleware, virtualization middleware design must support both the service providers and infrastructure providers to meet fluctuating and impulsive services and resource demands.
- Processing limitations of the sensor node, and high computational requirements for middleware supporting virtualization is an immense challenge.
- Lack of standardization is another key challenge there is still no consensus on the software and hardware platforms and heterogeneity keeps on growing.
- The middleware for virtualization should support widespread networks of wireless sensors using distinct radios and deal with the integration problem of the heterogeneous WSN.
- The virtualization middleware should be fault tolerant and adaptable to multiple network requirements.
- Virtualization middleware design should separate application dependent services from the network dependent services.
- Redundancy in virtualization has to be eliminated for efficient network operations.
- Efficient localization and clustering techniques in middleware for virtualization are required so as to reduce processing overhead.
- Middleware must incorporate new efficient algorithms for delay tolerance, load balancing and errors minimization, the conventional techniques will not work in the virtualized environment efficiently.

Heterogeneity, reliability, adaptability, re-usability, scalability, QoS and context awareness techniques for the virtualized environment need significant improvement and are the other key issues of a middleware design for WSN virtualization. Therefore, demand of lightweight design of a middleware that address the fundamental challenges identified above for virtualization of sensor network and IoE to be a reality. Even though numerous constraints make these problems computationally intractable, in the presence of multiple networks, topologies, links, services and applications provide opportunities to exploit and leave enough room for researchers to modify solutions, techniques and algorithms. All of the above mentioned challenges need to be addressed by the research community as they are the key to the future WSN virtualization and building blocks of IoE.

## 9. Major Components of Proposed Virtualization Model

When application directly communicates with the hardware without management of the services and the hardware, it usually results in wastage of the resources. Middleware is responsible for all the management of process between the application and the hardware. Virtualization middleware consist of a number of components few of the major components of the proposed middleware are discussed in this section. The services offered by the proposed middleware at the gateway and the node are of two types, the application dependent services and the network dependent services.

First of all, in middleware design, programming models are to be taken under consideration. Consequently, none of programming models alone seem to promise a generic programming solution and non can efficiently support and manage the sensor network. This investigation has motivated us to consider a hybrid programming model that is event based and utilizes modules that can be invoked whenever they are required according to the context. Virtualization middleware requirements match well with the publish/subscribe communication mode, as WSN are an event-based system. The middleware design utilizing the hybrid programming model will be more concerned with the resources of the sensor network.

The proposed programming model is based on the macro programming approach, providing a high level abstraction of the low level details. The modular approach is one of the key driving force, in the making the virtualization of networks successful. The component based model is used that supports SOA. Furthermore, an event based modular approach is used that also supports the asynchronous execution models. Thread abstraction is more expensive at the sensor node level as we have to dedicate a stack per thread that consumes a lot of memory. However, for real time applications and federated WSN multi-threading technique reduces the processing time quite significantly and the gateway being a powerful node with less memory constraints uses multi-threading model in the proposed middleware.

Middleware manages all the service provisioning stages between the user/application and the WSN nodes. Albeit, the data is managed well by virtualization, but checking all the parameters, delay the process, so the middleware components should be invoked in such a way that the latency is reduced as much as possible. Software model of the proposed middleware is based on Non-blocking and split phase techniques. Moreover, imperative programming components ensure the QoS. However, proposed middleware also provides few components supporting declarative commands to provide high level abstraction and ease of programming for the developer.

**Figure 5.**
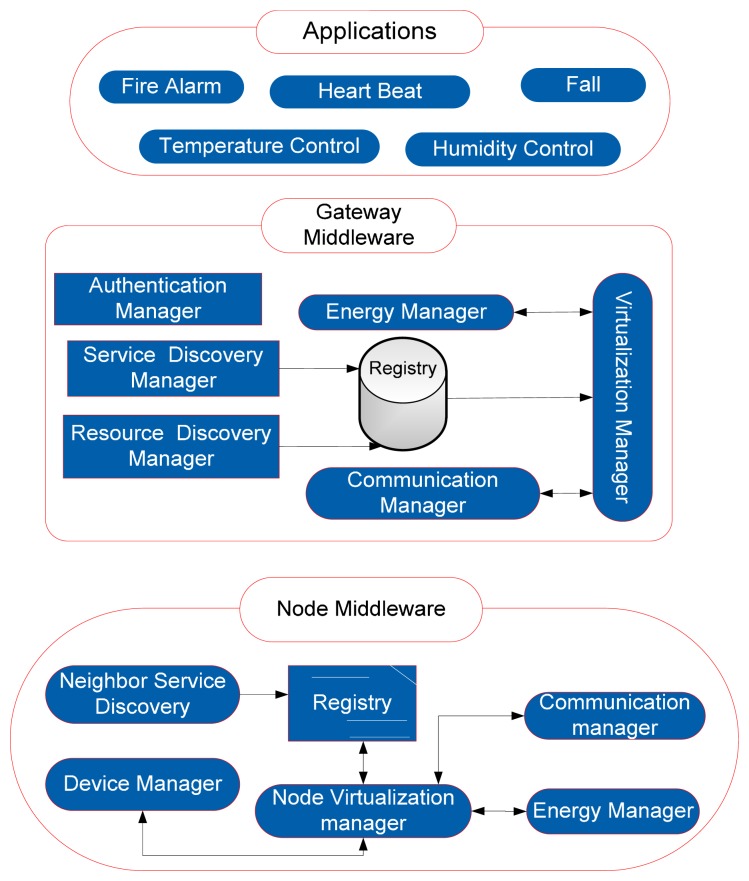
Proposed middleware design.

The event driven model is more suitable for microcontrollers as of their sleep nature, and can also achieve energy-efficiency. Figure 5 shows that the major components of application and network dependent services and their interaction. The proposed middleware design, resides at the gateway as well as the sensor node.

Service discovery is one of the key modules of the middleware design. It resides both on the gateway and the sensor node in order to efficiently provide and keep a record of all the services offered by the WSN. Service discovery module at the sensor node keeps the record of the one hop neighboring services and also updates the gateway about the services offered.

Resource discovery is more related to the hardware components and their capabilities such as how much battery life is left, the number of radios a node can support and the memory available, all these parameters are taken into consideration before forming a virtual network. It is more related to the infrastructure providers. Furthermore, this module keeps the record updated; it also resides at the node as well as the gateway. The device manager and the energy manager are the supporting components of the resource discovery module. The components like service discovery and resource discovery decouple the infrastructure from the services and applications.

Where services and infrastructure are shared, authentication plays a vital role. Similarly, in virtualized federated sensor networks, authentication manager makes sure the services and infrastructure provided should be accessed by the authenticated users and applications. The sensor device at home and the health care data in particular, should not be accessed by the unauthenticated user.

The virtualization manager is the brain of the middleware. It collaborates with energy manager, communication manager, resource discovery module, service discovery module and the registry to serve the user queries and application demands. Furthermore, it makes sure the application demands are met in an energy efficient manner. It resides at both the node and the gateway. The energy manager module takes the power parameters from different sensor nodes and manages the power of the network; it collaborates with the virtualization manager such that the nodes with more power are to be utilized intermittently, so as to increase the network longevity.

Communication manager is responsible for selecting the radios and the channels for communication among the nodes and the gateway. Application based radio selection makes sure that the application demands are fulfilled. The flexibility of switching between different radios provides high flexibility in terms of data rate and communication range. However, an eye has to be kept on the power consumption of transmission.

The middleware part that resides at the node consists of neighbor service discovery that keeps the record of the single hop neighbors' location, services they offer and the resources they have. Device manager module manages the sensor node hardware. It updates the virtualization manager about the malfunctioning, damage or tempering of any sensor attached to the node. The device manager and the energy manager modules enable nodes to update the gateway with their current state of power, radios and services. The node virtualization manager is connected to device manger, registry, energy manager and communication manager that allow virtual manager to manage all the node operations.

The gateway's virtualization manager along with its interfaces with the communication manager and registry tries to compute the best possible combination of clusters that satisfy the QoS demanded by the application. Furthermore, it also tries to accommodate the error and the delay in transmission of the data. Moreover, it tries to balance the load of traffic on the links and sensor nodes. The service discovery module along with the registry aggregates the data.

## 10. Conclusions

Middleware for virtualization presents a new potential research area. The importance of WSN virtualization is increasing day by day due to its capability of integrating heterogeneous devices, networks, radios and platforms. The study presented provides an overview of the middleware designs supporting sensor virtualization and network virtualization. The taxonomy of middleware presented is based on the support for the sensor virtualization, network virtualization and multi radio. The paper elaborates different programming paradigms and how they can be used for the middleware design based on virtualization of WSN. The research presents abstracted services and role in middleware designs. Furthermore, testbeds for middleware design evaluation and several future directions and challenges for designing the middleware. Major components of the proposed model are also presented that provide a general middleware design capable of handling both the sensor and network virtualization. Virtualization will gain massive popularity in the near future because of the upcoming trends of separation of sensor service providers from sensor infrastructure providers. The middleware will be the key in making the business successful by lowering costs for the end users, managing and decoupling the infrastructure and service providers.

## Figures and Tables

**Table 1.** Middleware architecture design and WSN virtualization support.

**Name**	**Software Architecture Design**	**Sensor Virtualization**	**Network Virtualization**	**Multi Radio Support**	**Heterogeneity Support**
Servilla [93]	Virtual Machine Service Oriented Architecture Platform independent applications execution	YES	NO	NO	YES
Melete [46]	Virtual machine Provides instruction set for sensors programming Modular system update mechanism Group-keyed programming model	YES	NO	NO	NO
Mate [21]	Virtual machine Provides instruction set for sensors programming 24 Byte instruction-long Instructions Split Phase non-blocking execution	YES	NO	NO	NO
SenShare [20]	Overlay Layer Hardware Abstraction Layer at Node Embedded Linux Multi-Task OS Split-Phase access	YES	YES	NO	NO
VITRO [44]	Component based Architecture Software Oriented Architecture Resource Oriented Architecture Virtualized modular mechanism	YES	YES	NO	NO
I-Living [94]	Application Driven architecture JAVA - API, SOAP based design TCP/IP	NO	YES	YES	NO
ROA based MW Framework [76]	ROA following SWE and modeled Sensor ML	YES	NO	YES	NO
MiLAN [75]	Adaptive System TCP/IP Graph based Model Application Centric Design	NO	YES	YES	NO
Smart Home Virtualization model [32]	Hardware Abstraction Layer at Gateway Embedded Linux Multi-Task OS Service Oriented Architecture	YES	YES	NO	NO
PRESTO [50]	Data Base approach Predictive Storage Architecture Caching mechanism	YES	NO	NO	YES
SenseWrap [69]	SOA UDP/TCP Zeroconf plugin Protocol Adapters	YES	YES	NO	YES
GSN [28]	Data Base Approach Data Oriented Approach SQL Based Query Declarative programming Model Container-based architecture	NO	YES	NO	YES
Hourglass [57]	SOA Circuit Description Language TCP/IP Publish Subscribe Approach	YES	YES	NO	NO
SENSEI [67]	Service Oriented Architecture Modular approach RESTful Design Layered approach Dynamic service composition	YES	YES	NO	NO

**Table 2.** Middleware and devices used for implementation.

**Middleware**	**Servilla [93]**	**Melete [46]**	**SenShare [20]**	**ROA Middleware [76]**	**Smart Home [32]**	**SenseWrap [69]**	**GSN [28]**	**SensEye [68]**	**MAMA [62]**
**Devices**	Imote2 TelosB	TelosB	Imote2	JN51XX	Imote2	Sun Spot	MIica2	Cyclops Crossbow Motes mini-ITX	Mica2

**Table 3.** Testbeds for evaluation of middleware designs.

**Testbed**	**Device Used**	**Number of Sensor Nodes**	**Services Offered**	**Virtualization Support**
University of Lübeck Smart Stender [141]	iSense TelosB Pacemate	200	Environment Light Security Accelerometer	Yes
Freie Universität Berlin [162]	DES-WSN DES-Mesh DES-WMNDEC-Mobile	110	TemperatureHumidity	Yes
Braunschweig Institute of Technology [163]	iSense	30 Nodes 120 Sensors	Load SensorsWeight	Yes
Research Academic Computer Technology Institute [164]	iSense TelosB	154	Environment SecurityLight Control	Yes
Universitat Politecnica de Catalunya [165]	iSense	10	Solar module TemperatureLight	Yes
Universität Bern [166]	TelosBMSB-430	47	Temperature Humidity LightAccelerometer	Yes
University of Geneva [167,168]	Atom-Based Servers iSense	25	Security Target Tracking	Yes
Delft University of Technology [169,170]	SOWNet T-Node SOWNet G-Node Tmote Sky TelobsB Octopus I-II MICAz	140	Temperature Humidity	Yes
Lancaster University [171]	TelosB	16	Temperature Humidity	Yes
University of Cyprus [172]	MICAz Camera	14	Environment Chemical Mobility	No
University of Thessaly OneLab/Open Lab [173–175]	GNU-MIMO Diskless	80	Cameras Temperature Humidity	Yes
KAIST University SmartFIRE [161]	MTS300 MTS420 MPR2400 MIB600	50	Temperature Acoustic	Yes
Washington University [176]	TelosB	79	Light Radiation Temperature Humidity	No
National University of Singapore [177,178]	TelosB	139	Light Temperature	No
TARWIS [166,179]	TelosB	200	Environment	No
NetEye [180]	TelosB	130	Light Environment	No
SmartSantander	TelosB RFID	20,000	Temperature CO Light	Yes
Technische Universitat Berlin TWIST [160]	TelosB eyesIFX	204	Light Temperature	No
HOBNETUNIGE [181]	TelosB iSense	20	Temperature Humidity Electric Device Monitoring	Yes
IoT-LAB [161]	WSN-430 M3 A8	2700	Temperature Light Environment	Yes
University of CambridgeSenShare [20]	iMote 2	35	Temperature Light Humidity Acceleration	Yes
